# Supplementary material for: PLCγ-dependent mTOR signalling controls IL-7-mediated early B cell development
Source: Nat Commun. 2017 Nov 13;8:1457. doi: 10.1038/s41467-017-01388-5 (PMC5684131; doi:10.1038/s41467-017-01388-5)
Supplement: Supplementary file 1 — Supplementary information [file 41467_2017_1388_MOESM1_ESM.pdf]

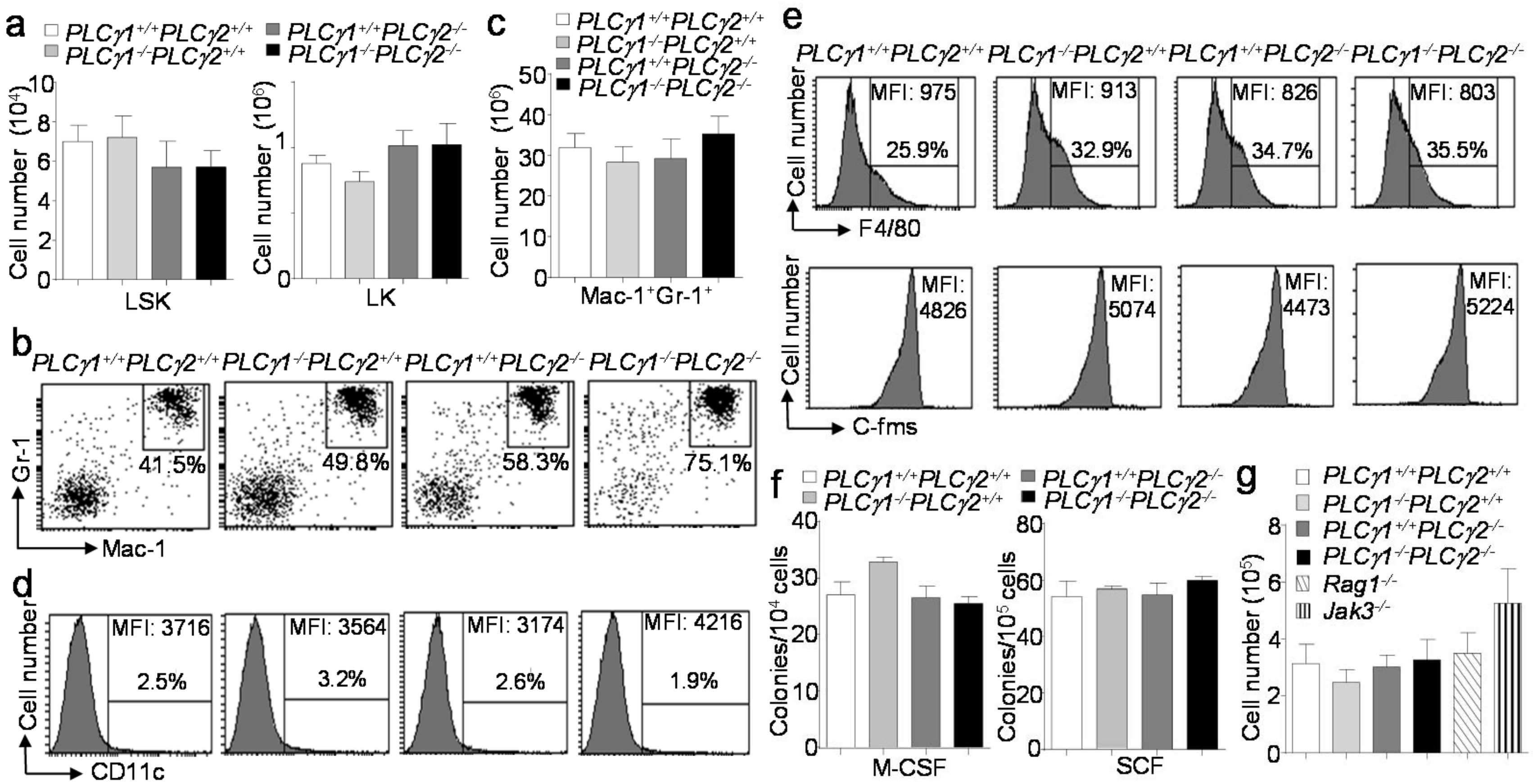
a
PLCγ1+/+PLCγ2+/+
PLCγ1-/-PLCγ2+/+
PLCγ1+/+PLCγ2-/-
PLCγ1-/-PLCγ2-/-
Cell number (10^4)
LSK
Cell number (10^6)
LK
c
Cell number (10^6)
Mac-1+Gr-1+
b
Gr-1
Mac-1
41.5%
49.8%
58.3%
75.1%
d
Cell number
CD11c
MFI: 3716
2.5%
MFI: 3564
3.2%
MFI: 3174
2.6%
MFI: 4216
1.9%
e
Cell number
F4/80
MFI: 975
25.9%
MFI: 913
32.9%
MFI: 826
34.7%
MFI: 803
35.5%
C-fms
MFI: 4826
MFI: 5074
MFI: 4473
MFI: 5224
f
Colonies/10^4 cells
M-CSF
Colonies/10^5 cells
SCF
g
Rag1-/-
Jak3-/-
Cell number (10^5)

**Supplementary Figure 1.**

**The effect of *PLCγ1/PLCγ2* double deficiency on hematopoietic progenitors and myeloid cells.** BM from poly(I-C)-treated *PLCγ1*$^{+/+}$*PLCγ2*$^{+/+}$, *PLCγ1*$^{-/-}$*PLCγ2*$^{+/+}$, *PLCγ1*$^{+/+}$*PLCγ2*$^{-/-}$ or *PLCγ1*$^{-/-}$*PLCγ2*$^{-/-}$ mice were transplanted into lethally irradiated congenic wild-type CD45.1$^{+}$ mice. Six to eight weeks after transplantation, BM cells from the recipients received the indicated donor BM were analyzed. (a) Bar graphs show the numbers of LSK (CD45.2$^{+}$Lin$^{-}$IL-7R$^{-}$Sca1$^{+}$c-Kit$^{+}$) and LK (CD45.2$^{+}$Lin$^{-}$IL$^{-}$7R$^{-}$Sca1$^{-}$c-Kit$^{+}$) cells. Error bars show ± SEM. (b) Percentages of Mac-1$^{+}$Gr-1$^{+}$ cells in the gated CD45.2$^{+}$ population. (c) Bar graphs show the numbers of CD45.2$^{+}$Mac-1$^{+}$Gr-1$^{+}$ cells. Error bars show ± SEM. (d,e) Histograms show percentages of CD11c$^{+}$ (d) and F4/80$^{+}$ (upper) and c-fms$^{+}$ (lower) (e) in the gated myeloid population. (f) BM cells from the recipients were plated in duplicate with M-CSF or SCF. Colonies were scored at day 12. (g) Lin$^{-}$ BM cells from the recipients, *Rag1*$^{-/-}$ or *Jak3*$^{-/-}$ mice were cultured on OP9 cells in the presence of SCF. The cells were stained with anti-CD45.2 and anti-Mac-1 after 9 days of co-culture. Bar graphs show the numbers of CD45.2$^{+}$Mac-1$^{+}$ cells. Data shown are obtained from 12 (a), 11 (c) or 8 (g) mice of each genotype or representative of 11 (b), 4 (d,e) or 2 (f) independent experiments.

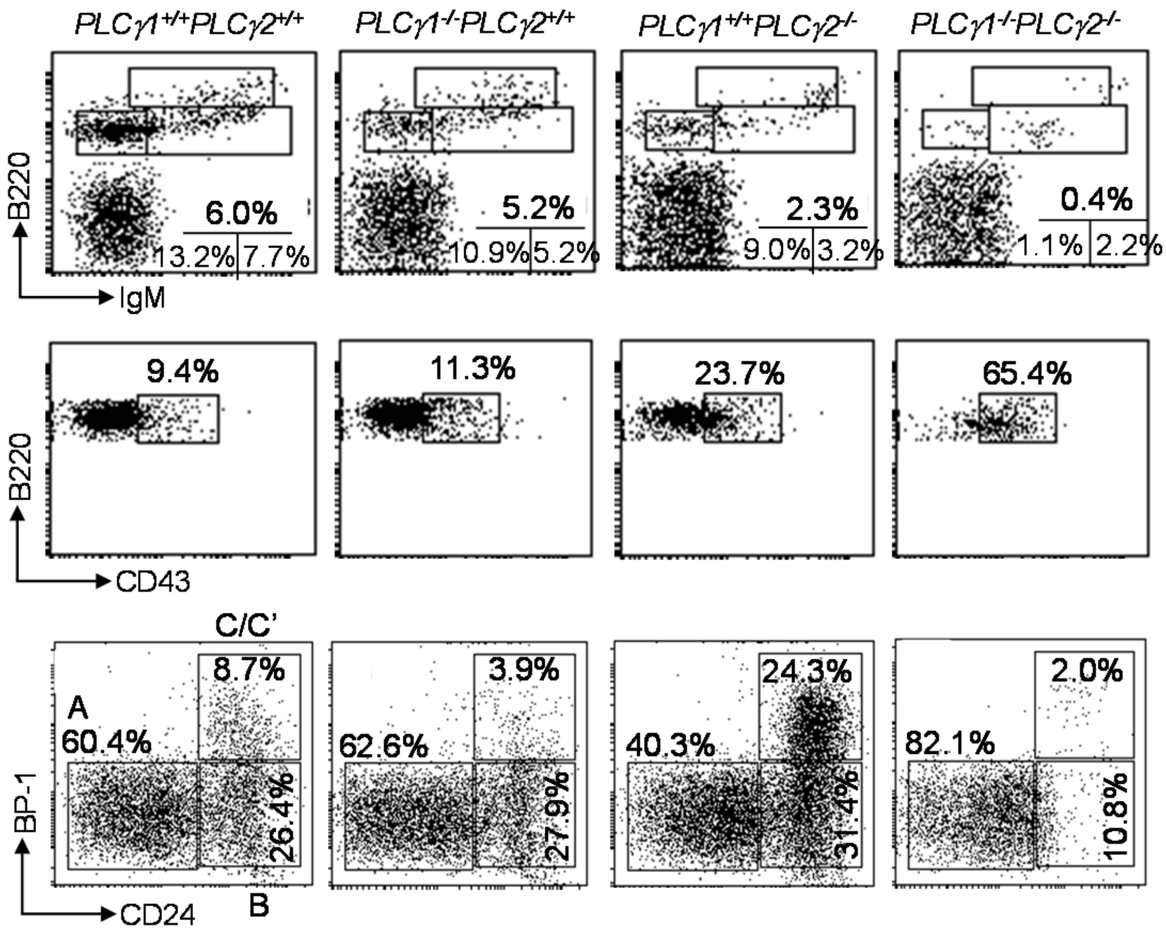


**Supplementary Figure 2.**

***PLCγ1/PLCγ2* double deficiency blocks B cell development at the pre-pro-B cell stage in nontransplanted mice.** Three weeks after the last poly (I-C) injection, BM cells from *PLCγ1*$^{+/+}$*PLCγ2*$^{+/+}$, *PLCγ1*$^{-/-}$*PLCγ2*$^{+/+}$, *PLCγ1*$^{+/+}$*PLCγ2*$^{-/-}$ or *PLCγ1*$^{-/-}$*PLCγ2*$^{-/-}$ mice were stained with anti-B220, anti-IgM, anti-CD43, anti-CD24 and anti-BP-1. Numbers indicate percentages of pro/pre-, immature and mature B cells in the gated YFP$^{+}$ live cell population (upper), percentages of pro-B cells in the gated B220$^{+}$IgM$^{-}$ population (middle), and percentages of pre-pro-B (fraction A), early pro-B (Fraction B) and late pro-B/early pre-B (fraction C/C') cells in the gated B220$^{+}$CD43$^{+}$ pro-B cell population (lower). Data shown are representative of 3 mice of each genotype.

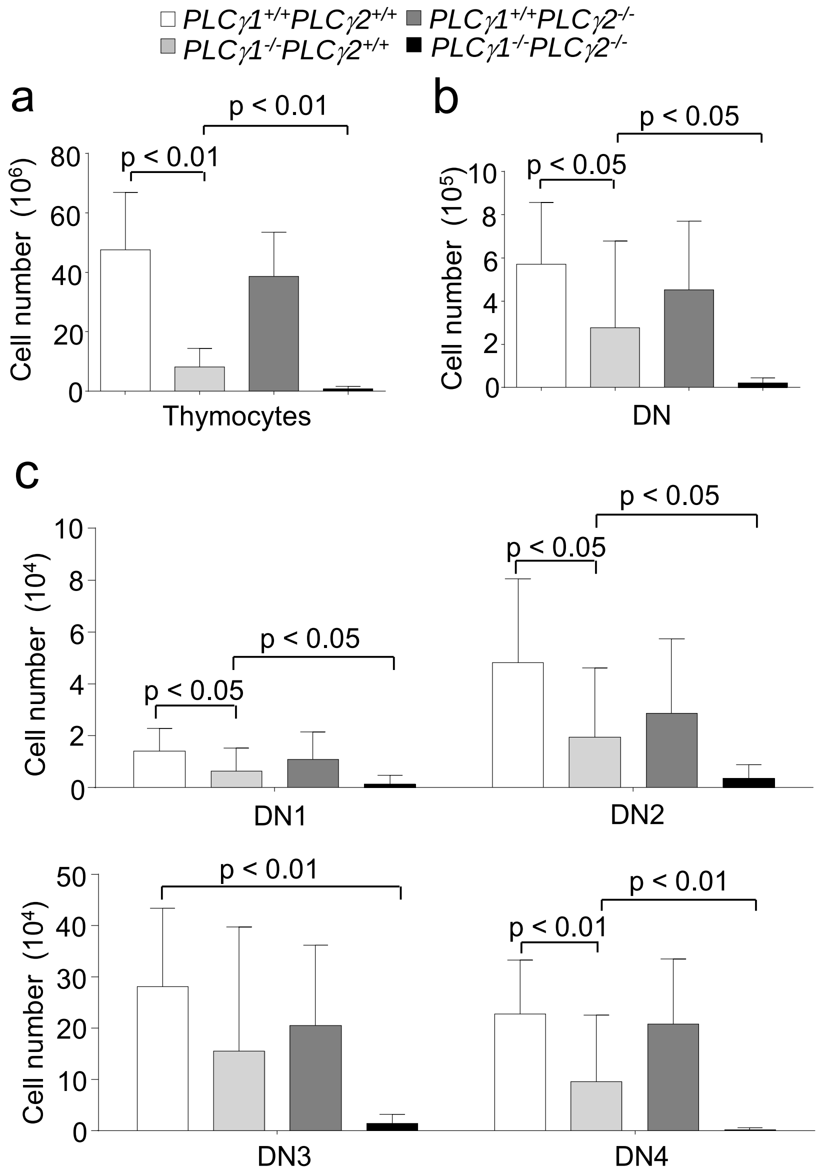


**Supplementary Figure 3.**

***PLCγ1/PLCγ2* double deficiency blocks T cell development at DN stage.** BM from poly(I-C)-treated $PLC\gamma1^{+/+}PLC\gamma2^{+/+}$, $PLC\gamma1^{-/-}PLC\gamma2^{+/+}$, $PLC\gamma1^{+/+}PLC\gamma2^{-/-}$ or $PLC\gamma1^{-/-}PLC\gamma2^{-/-}$ mice were transplanted into lethally irradiated congenic wild-type $CD45.1^{+}$ mice. Six to eight weeks after transplantation, thymocytes from the recipients were analyzed. Bar graphs show the numbers of total thymotyes (a), $CD45.2^{+}Lin^{-}$($B220^{-}CD11b^{-}Gr.1^{-}NK1.1^{-}$) DN ($CD4^{-}CD8^{-}$) (b), and DN1 ($CD4^{-}CD8^{-}CD44^{+}CD25^{-}$), DN2 ($CD4^{-}CD8^{-}CD44^{+}CD25^{+}$), DN3 ($CD4^{-}CD8^{-}CD44^{-}CD25^{+}$) and DN4 ($CD4^{-}CD8^{-}CD44^{-}CD25^{-}$) (c) thymocytes of the recipients received indicated donor BM. Error bars show ± SEM. Data shown are obtained from 16 $PLC\gamma1^{+/+}PLC\gamma2^{+/+}$, 14 $PLC\gamma1^{-/-}PLC\gamma2^{+/+}$, 8 $PLC\gamma1^{+/+}PLC\gamma2^{-/-}$ and 15 $PLC\gamma1^{-/-}PLC\gamma2^{-/-}$ mice.

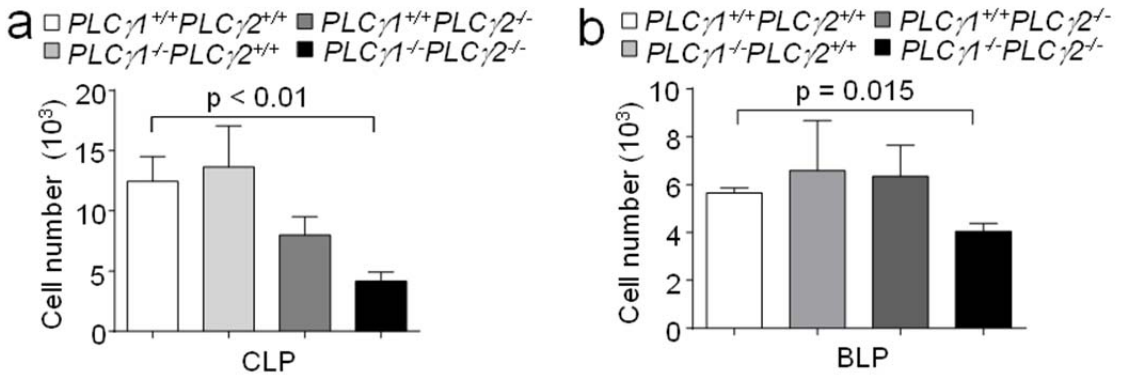


**Supplementary Figure 4.**

***PLCγ1/PLCγ2* double deficiency results in reduction of CLPs and BLPs.** BM from poly(I-C)-treated *PLCγ1*$^{+/+}$*PLCγ2*$^{+/+}$, *PLCγ1*$^{-/-}$*PLCγ2*$^{+/+}$, *PLCγ1*$^{+/+}$*PLCγ2*$^{-/-}$ or *PLCγ1*$^{-/-}$*PLCγ2*$^{-/-}$ mice were transplanted into lethally irradiated congenic wild-type CD45.1$^{+}$ mice. Six to eight weeks after transplantation, BM cells from recipient mice were analyzed by flow cytometry. Bar graphs showed the cell numbers of CLP (CD45.2$^{+}$Lin$^{-}$IL$^{-}$7R$^{+}$Sca1$^{med}$c-Kit$^{med}$) (a) and BLP (CD45.2$^{+}$Lin$^{-}$ IL$^{-}$7R$^{+}$Sca1$^{med}$c-Kit$^{med}$ Ly6D$^{+}$) (b) populations in the BM of the recipients received the indicated donor BM. Error bars show ± SEM. Data shown are obtained from 4 (a) or 3 (b) mice of each genotype.

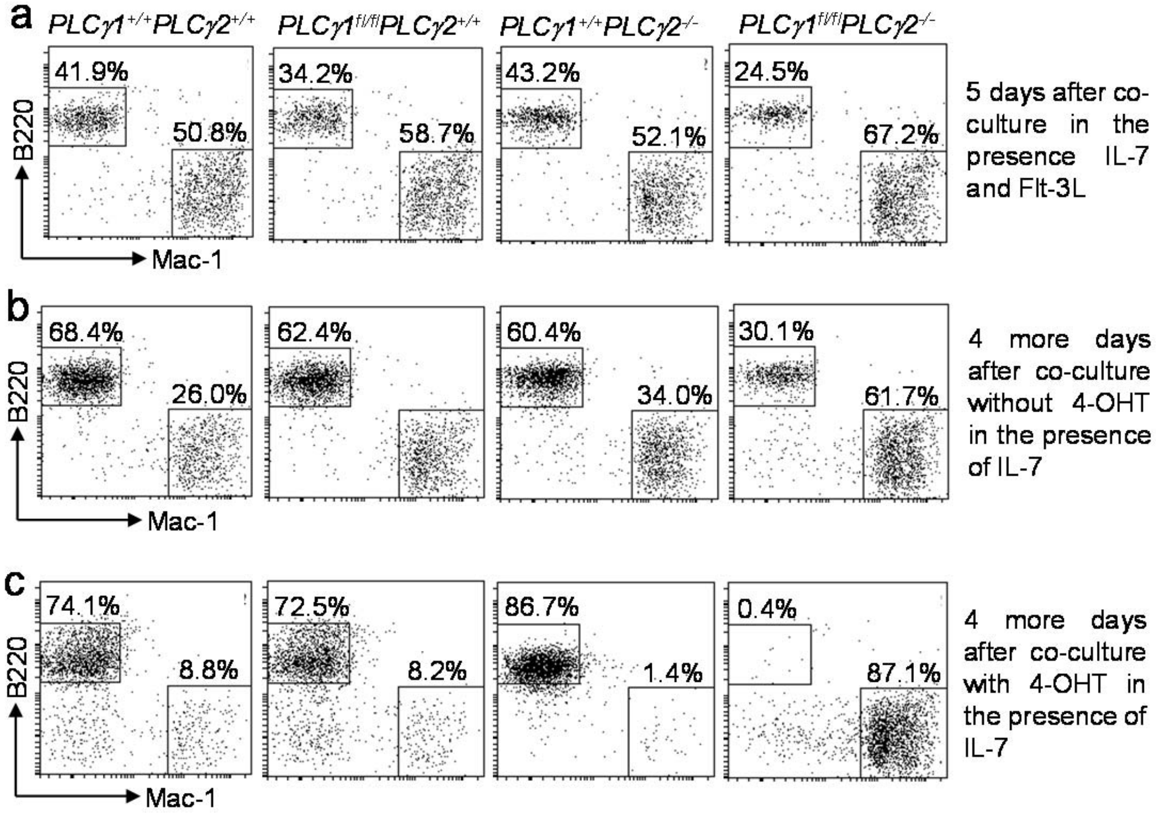


**Supplementary Figure 5.**

***A*cute deletion of the both *PLCγ1* and *PLCγ2* genes in vitro inhibits IL-7-dependent B cell production.** BM from *ERCre PLCγ1*$^{+/+}$*PLCγ2*$^{+/+}$, *ERCrePLCγ1*$^{fl/fl}$*PLCγ2*$^{+/+}$, *PLCγ1*$^{+/+}$*PLCγ2*$^{-/-}$ or *ERCrePLCγ1*$^{fl/fl}$*PLCγ2*$^{-/-}$ mice were transplanted into lethally irradiated congenic wild-type CD45.1$^{+}$ mice. Six to 8 weeks after transplantation, Lin$^{-}$ BM cells from the recipients mice were cultured on OP9 cells in the presence of IL-7 and Flt3L for 5 days (a) and then 1x $10^5$ cells from each genotype were further cultured on OP9 in the presence of IL-7 without (b) or with (c) 4-OHT for 4 days. The cells were stained with anti-CD45.2, anti-B220 and anti-Mac-1. Numbers indicate percentages of B (B220$^{+}$) and myeloid (Mac-1$^{+}$) cells in the gated CD45.2$^{+}$ population. Data shown are representative of 2 independent experiments.

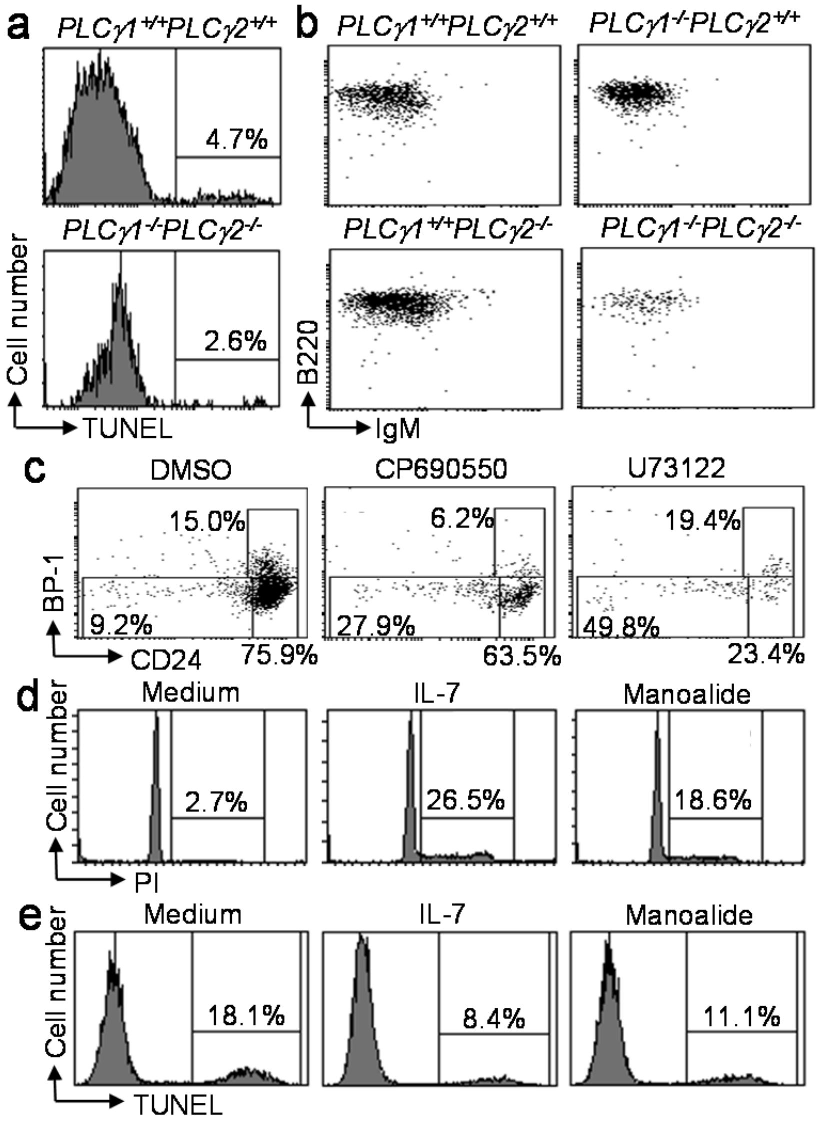


**Supplementary Figure 6.**

**Role of the PLCγ pathway in IL-7-induced B cell production, proliferation and survival.** (a,b) *PLCγ1/γ2* double deficiency impairs IL-7-mediated production but not survival of B cell progenitors. (a) $YFP^+CD45.2^+B220^+CD43^+CD24^+$ B cell progenitors were sorted from the recipients of *PLCγ1*$^{+/+}$*PLCγ2*$^{+/+}$ or *PLCγ1*$^{-/-}$*PLCγ2*$^{-/-}$ BM, and co-cultured with OP9 cells in and IL-7 for 3 days. Cell survival was measured by TUNEL staining. Numbers indicate percentages of $TUNEL^+$ cells among these B cell progenitors. (b) *PLCγ1*$^{+/+}$*PLCγ2*$^{+/+}$, *PLCγ1*$^{-/-}$*PLCγ2*$^{+/+}$, *PLCγ1*$^{+/+}$*PLCγ2*$^{-/-}$ or *PLCγ1*$^{-/-}$*PLCγ2*$^{-/-}$ BM were cultured with IL-7 alone. After 7 days, the cells were stained with anti-CD45.2, anti-B220 and anti-IgM and B220 and IgM staining of $CD45.2^+$ gated cells is shown. (c-e) Inhibition of the PLCγ pathway impairs IL-7-induced B cell production, proliferation and survival. (c) $Lin^-$ BM cells from wild-type mice were cultured on OP9 cells with IL-7 for 1 day and the cells were then cultured with DMSO, CP690550 (0.1 mM) or U73122 (0.03 mM). Four days later, the cells were stained with anti-B220, anti-CD43, anti-

CD24 and anti-BP-1. CD24 and BP-1 staining of $B220^+CD43^+$ gated cells is shown. Numbers indicate percentages of pre-pro-B (Fraction A), early pro-B (Fraction B) and late pro-B/early pre-B (Fraction C/C') cells in the gated $B220^+CD43^+$ cells. (d,e) BM cells from wild-type mice were cultured in the presence of IL-7 for 7 days to derive B cell progenitors. Then, these B cell progenitors were cultured without IL-7 (medium) or with IL-7 plus DMSO or Manoalide. Cell cycle analysis was performed by PI staining (d) and cell survival was measured by TUNEL staining (e). Data shown are representative of 2 (a,b), 3 (c) or 4 (d,e) independent experiments.

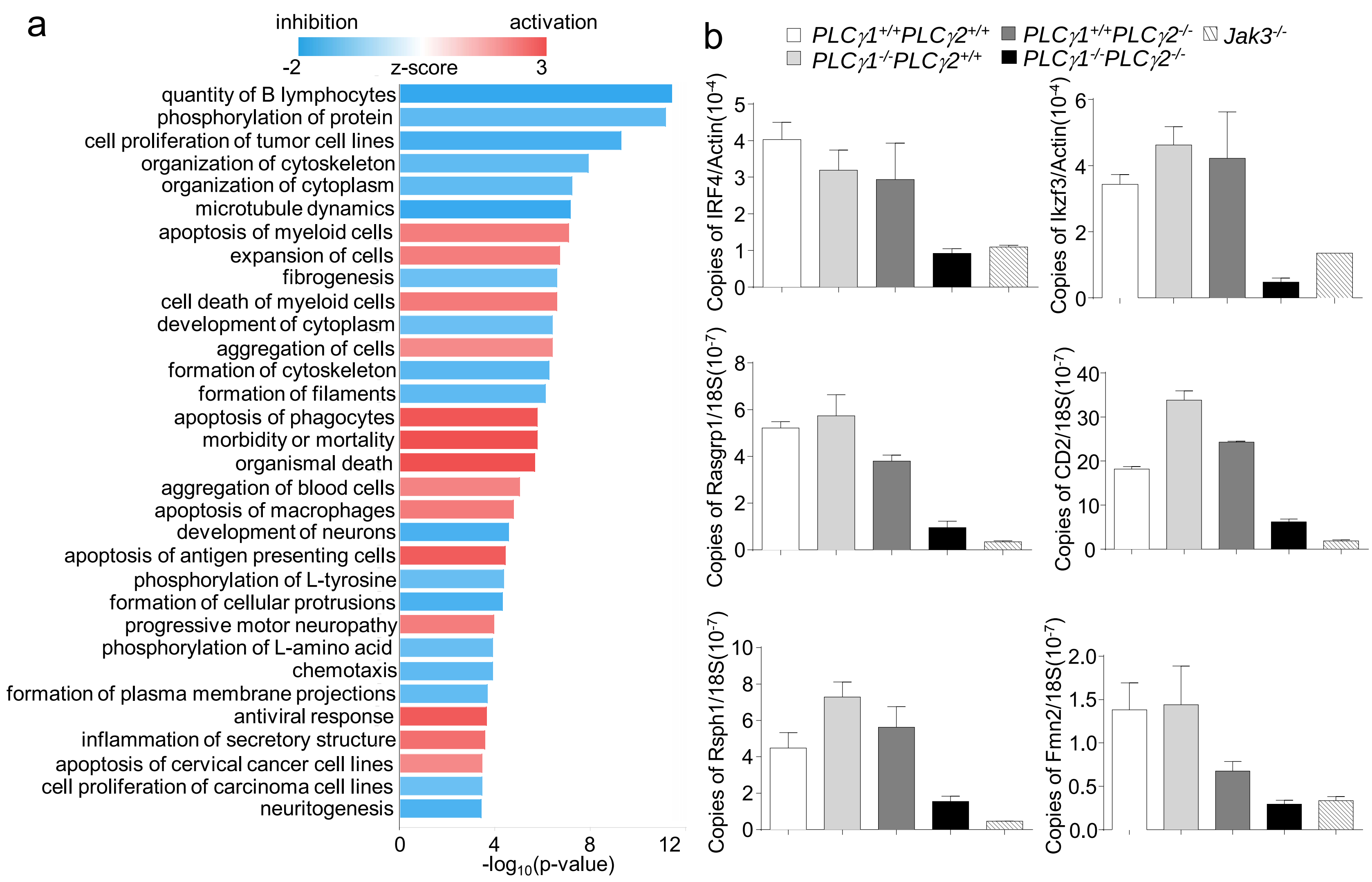
a
inhibition
activation
-2
z-score
3
quantity of B lymphocytes
phosphorylation of protein
cell proliferation of tumor cell lines
organization of cytoskeleton
organization of cytoplasm
microtubule dynamics
apoptosis of myeloid cells
expansion of cells
fibrogenesis
cell death of myeloid cells
development of cytoplasm
aggregation of cells
formation of cytoskeleton
formation of filaments
apoptosis of phagocytes
morbidity or mortality
organismal death
aggregation of blood cells
apoptosis of macrophages
development of neurons
apoptosis of antigen presenting cells
phosphorylation of L-tyrosine
formation of cellular protrusions
progressive motor neuropathy
phosphorylation of L-amino acid
chemotaxis
formation of plasma membrane projections
antiviral response
inflammation of secretory structure
apoptosis of cervical cancer cell lines
cell proliferation of carcinoma cell lines
neuritogenesis
0
4
8
12
$-\log_{10}$(p-value)
b
$PLC\gamma1^{+/+}PLC\gamma2^{+/+}$
$PLC\gamma1^{-/-}PLC\gamma2^{+/+}$
$PLC\gamma1^{+/+}PLC\gamma2^{-/-}$
$PLC\gamma1^{-/-}PLC\gamma2^{-/-}$
$Jak3^{-/-}$
Copies of IRF4/Actin($10^{-4}$)
Copies of Ikzf3/Actin($10^{-4}$)
Copies of Rasgrp1/18S($10^{-7}$)
Copies of CD2/18S($10^{-7}$)
Copies of Rsph1/18S($10^{-7}$)
Copies of Fmn2/18S($10^{-7}$)

**Supplementary Figure 7.**

***PLCγ1/PLCγ2* double deficiency alters gene expression in B cell progenitors.** (a) Top activated and inhibited pathways in *PLCγ1*$^{-/-}$*PLCγ2*$^{-/-}$ pro-B cells. Genes differentially expressed (FDR < 0.05) in *PLCγ1*$^{-/-}$*PLCγ2*$^{-/-}$ relative to *PLCγ1*$^{+/+}$*PLCγ2*$^{+/+}$ pro-B cells from Fig 4 were analyzed using Ingenuity Pathways software. Top activated and inhibited pathways are shown. Bars are colored by Ingenuity z-score which estimates relative activation (positive z-score) or inhibition (negative z-score). (b) The reduction of some of the top listed genes from Figs 4d-g was confirmed by qRT-PCR. Data shown is representative of 3 independent experiments.

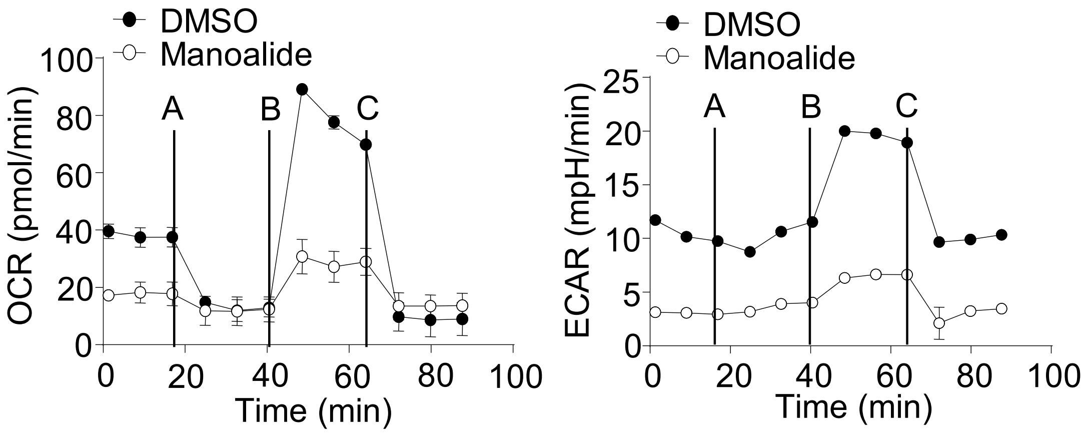


**Supplementary Figure 8.**

**Inhibition of the PLCγ pathway by Manoalide impairs IL-7-mediated metabolism of B cell progenitors.** $B220^{+}IgM^{-}IL\text{-}7R^{+}$ B cell progenitors were sorted from wild-type mice and cultured without IL-7 (medium) or with IL-7 plus DMSO or Manoalide. After measuring basal OCR (left) and ECAR (right), oligomycin A (A), FCCP (B) and antimycin (C) were sequentially added. Error bars show ± SEM. Data shown are obtained from 3 independent experiments.

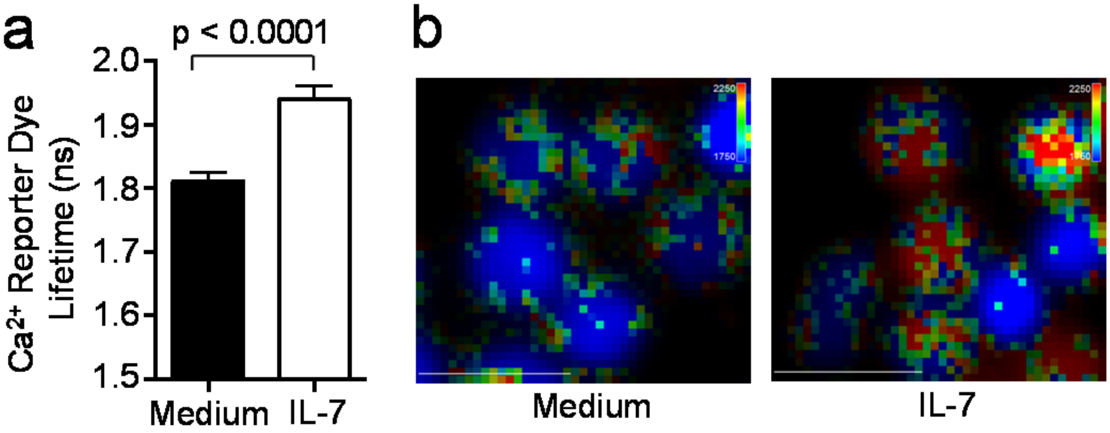


**Supplementary Figure 9.**

**Activation of the PLCγ pathway upon IL-7 stimulation.** Pro-B cells derived from Rag1-deficient BM were starved without IL-7 for 3 hours and then loaded with $Ca^{2+}$ reporter dye Oregon Green 488 BAPTA-2 for 1 hour. Cells were washed and plated into μ-Slides in the absence (Medium) or presence (IL-7) of IL-7 for 16 hours. Relative intracellular $Ca^{2+}$ levels were evaluated by fluorescence lifetime microscopy. (a) Average $Ca^{2+}$ reporter dye lifetime from analyzed cells (n = 75). Data are pooled from 2 independent experiments. (b) Representative lifetime images of $Ca^{2+}$ reporter dye signal. Blue and red colors were correlated with lower and higher $Ca^{2+}$ concentration, respectively.

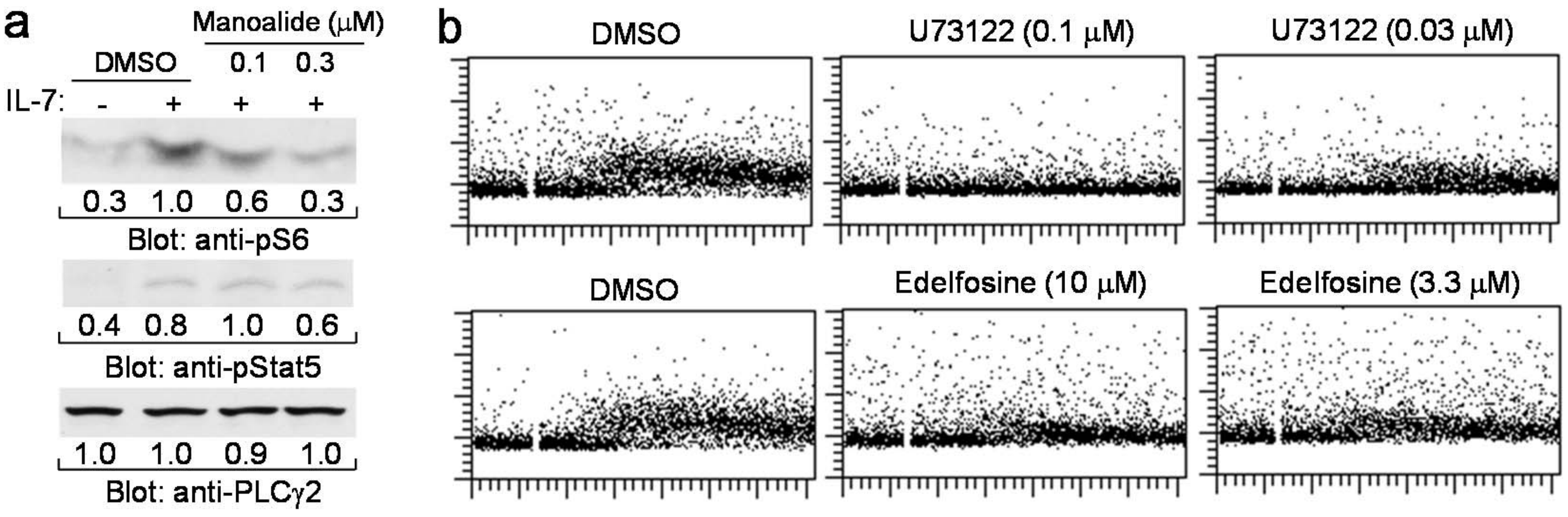
a
Manoalide (μM)
DMSO
0.1 0.3
IL-7: - + + +
0.3 1.0 0.6 0.3
Blot: anti-pS6
0.4 0.8 1.0 0.6
Blot: anti-pStat5
1.0 1.0 0.9 1.0
Blot: anti-PLCγ2
b
DMSO
U73122 (0.1 μM)
U73122 (0.03 μM)
DMSO
Edelfosine (10 μM)
Edelfosine (3.3 μM)

**Supplementary Figure 10.**

**PLCγ pathway inhibition impairs IL-7-induced mTOR activation in B cell progenitors and BCR-induced $Ca^{2+}$ flux in mature B cells.** (a) Inhibition of the PLCγ pathway impairs IL-7-induced mTOR activation in B cell progenitors. Pro-B cells derived from wild-type BM were pre-treated with DMSO or Manoalide and then stimulated with IL-7. Cell lysates were subjected to Western blot analysis with the indicated antibodies. Data shown are representative of 4 independent experiments. (b) Inhibition of the PLCγ pathway impairs BCR-induced $Ca^{2+}$ flux in mature B cells. Splenocytes from WT mice were labeled with Indo-1 and stained with anti-B220, anti-IgM and anti-IgD. The cells were treated with DMSO, U73122 or Edelfosine and then stimulated with anti-IgM, and $Ca^{2+}$ flux in mature B cells was measured by flow cytometry. Data shown are representative of 3 independent experiments.

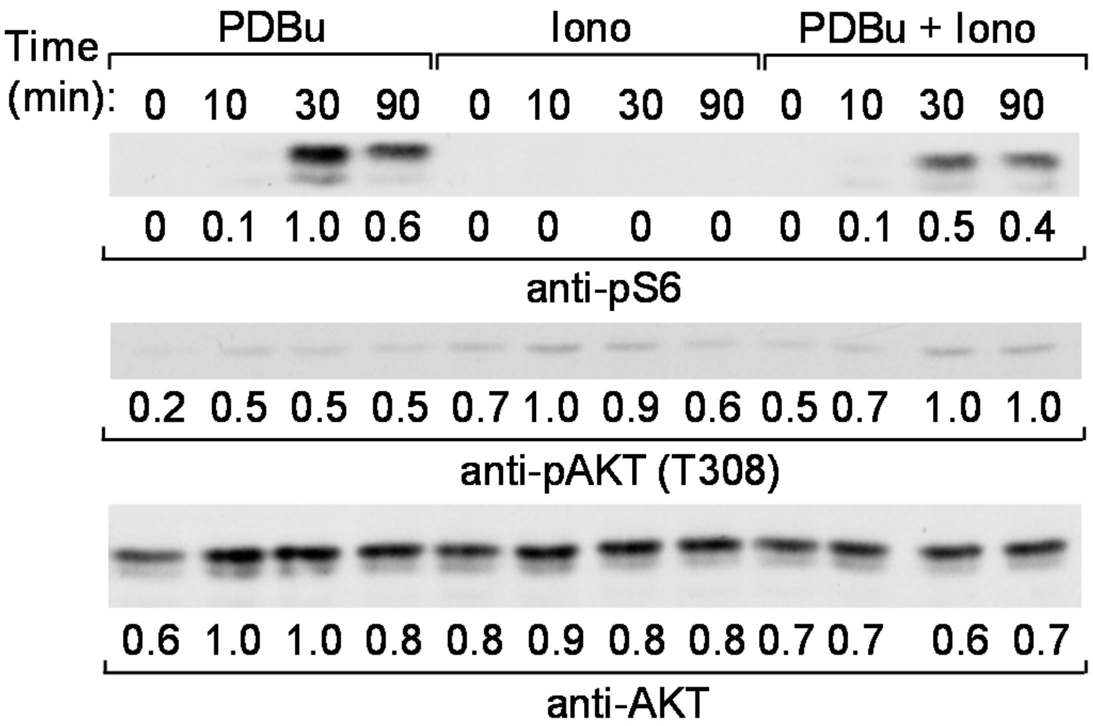


**Supplementary Figure 11.**

**The PKC pathway plays a minor role in Akt activation in pro-B cells.** Pro-B cells derived from *Rag1*-deficient BM were starved and stimulated with PDBu, ionomycin or PDBu plus ionomycin. Cell lysates were subjected to Western blot analysis with the indicated antibodies. Data shown are representative of 3 independent experiments.

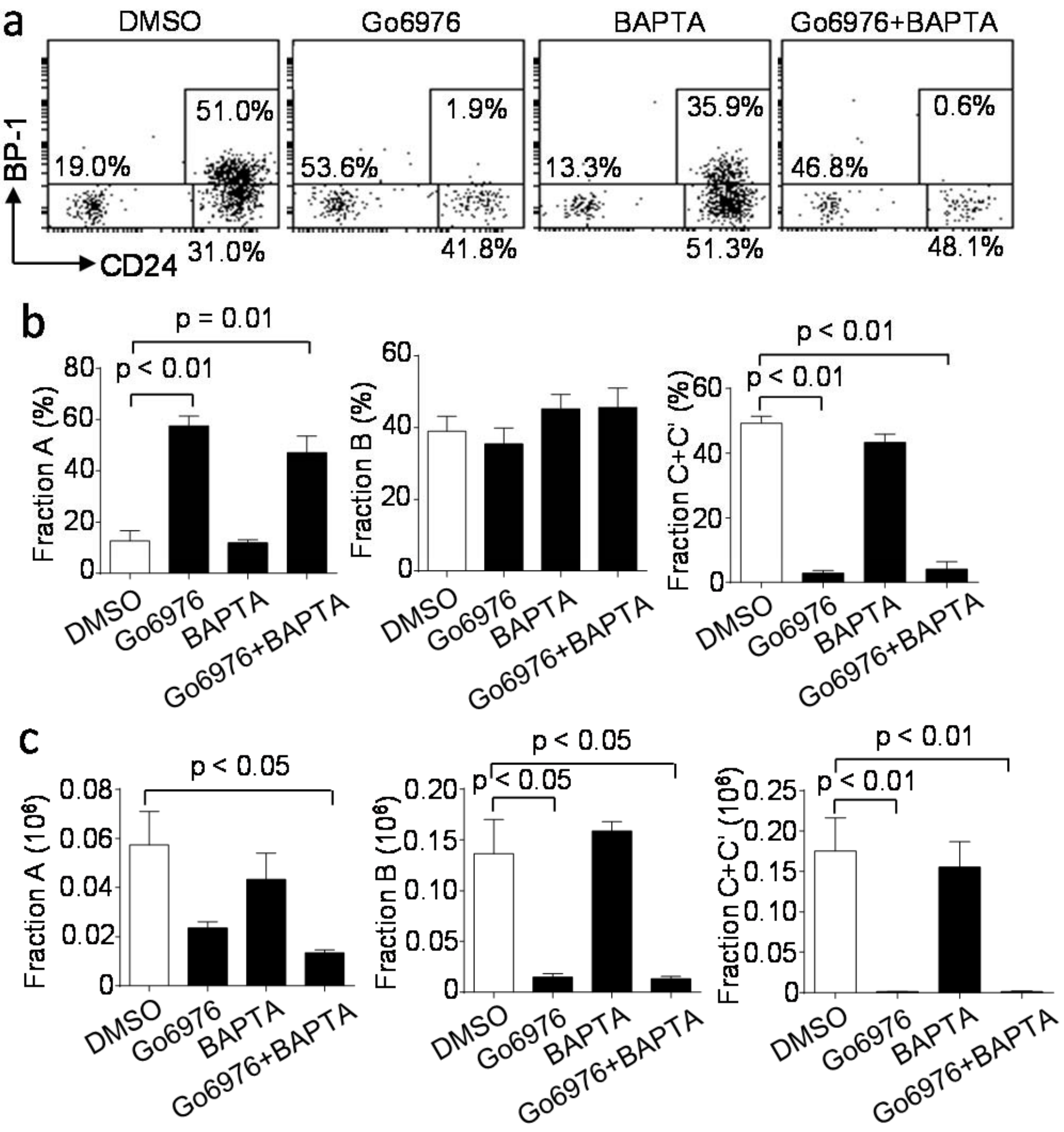


**Supplementary Figure 12.**

**PKC pathway inhibition impairs IL-7-dependent B cell development at the pre-pro-B cell stage in vitro.** $Lin^-$ BM cells from wild-type mice were cultured on OP9 cells in the presence of IL-7 for 1 day and then the cells were treated with DMSO, Go6976, BAPTA or Go6976 plus BAPTA. Four days later, the cells were stained with anti-B220, anti-CD43, anti-CD24 and anti-BP-1. Numbers indicate percentages of pre-pro-B (fraction A), early pro-B (Fraction B) and late pro-B/early pre-B (Fraction C/C') cells in the gated $B220^+CD43^+$ cells (a) and bar graphs show the percentages (b) and numbers (c) of fractions A, B and C/C' cells in the culture. Error bars show ± SEM. Data shown are obtained from or representative of 3 independent experiments.

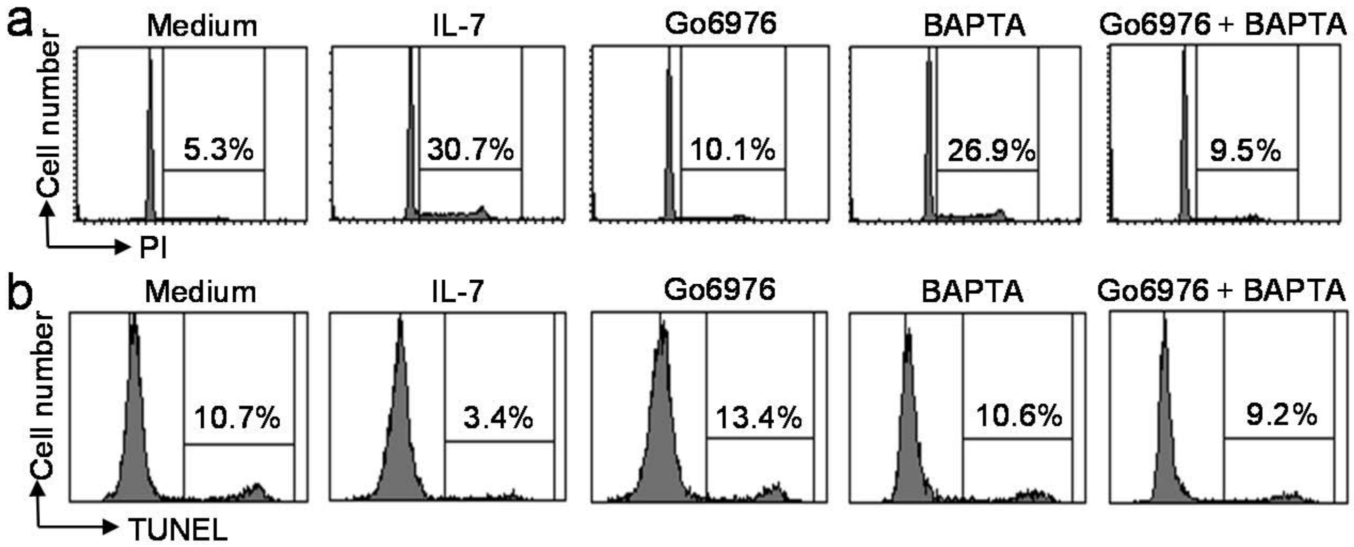


**Supplementary Figure 13.**

**Inhibition of the PKC pathway impairs IL-7-mediated proliferation and survival of unexpanded B cell progenitors.** B220$^{+}$IgM$^{-}$IL-7R$^{+}$ B cell progenitors were sorted from wild-type mice and cultured without IL-7 (medium) or with IL-7 and DMSO, Go6976, BAPTA or Go6976 plus BAPTA. Cell cycle analysis was performed by PI staining (a) and cell survival was measured by TUNEL staining (b). Data shown are representative of 5 independent experiments.

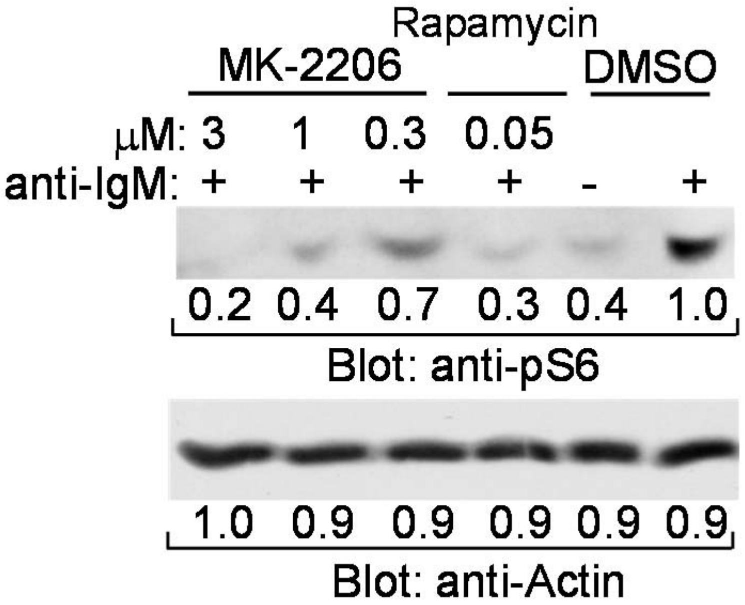


**Supplementary Figure 14.**

**AKT pathway inhibition impairs IgM-induced activation of mTOR.** Splenic B cells from wild-type mice were pre-treated with MK-2206, rapamycin or DMSO at the indicated concentration and stimulated with $F(ab')_2$ anti-IgM antibodies. Cell lysates were subjected to Western blot analysis with the indicated antibodies. Data shown are representative of 2 independent experiments.

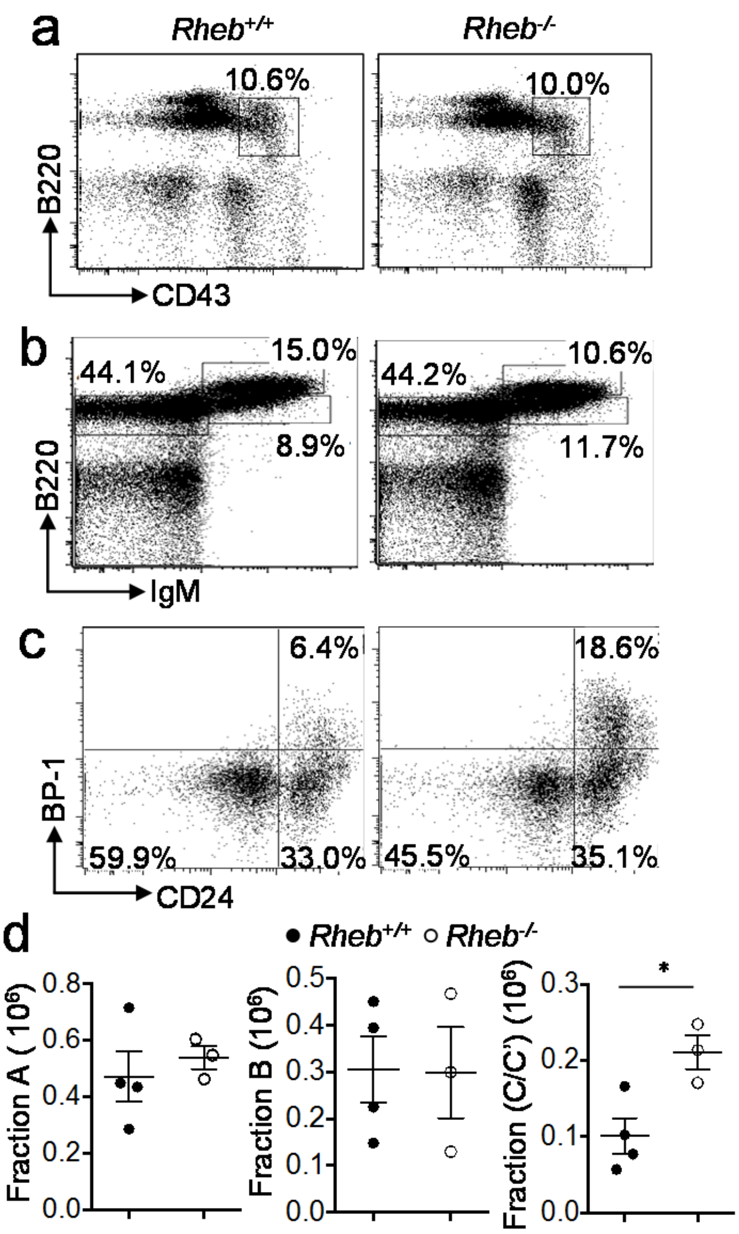


**Supplementary Figure 15.**

***Rheb* deficiency does not affect IL-7-mediated pre-pro-B to early pro-B cell transition.** BM cells from $Rheb^{+/+}$ and $Rheb^{-/-}$ mice were stained with anti-B220, anti-CD43, anti-BP-1, anti-CD24 and anti-IgM. Numbers indicate percentages of pro-B cells in the gated $B220^{+}$ population (a), percentages of pro/pre-, immature and mature B cells in the gated lymphoid population (b), and percentages of pre-pro-B (fraction A), early pro-B (fraction B) and late pro-B/early pre-B (fraction C/C') cells in the gated $B220^{+}CD43^{+}$ pro-B cell population (c). (d) Dot plots show the numbers of fractions A, B and C/C' cells. Each dot represents an individual mouse, horizontal bars indicate mean values, and error bars show ± SEM. Data shown are representative of or obtained from 4 *Rheb*$^{+/+}$ and 3 *Rheb*$^{-/-}$ mice.

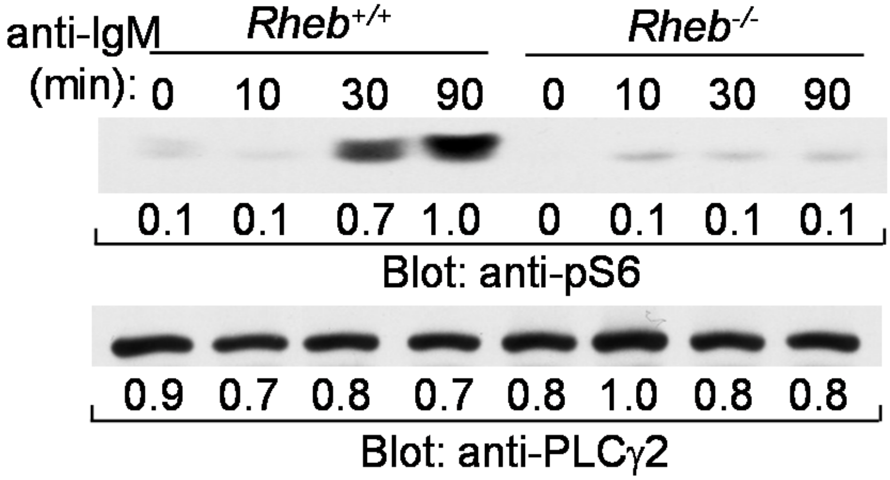


**Supplementary Figure 16.**

**BCR-induced mTOR activation is Rheb-dependent.** Splenic B cells from *Rheb*$^{+/+}$ and *Rheb*$^{-/-}$ mice were stimulated with anti-IgM. Cell lysates were subjected to Western analysis with the indicated antibodies. Data shown are representative of 2 independent experiments.

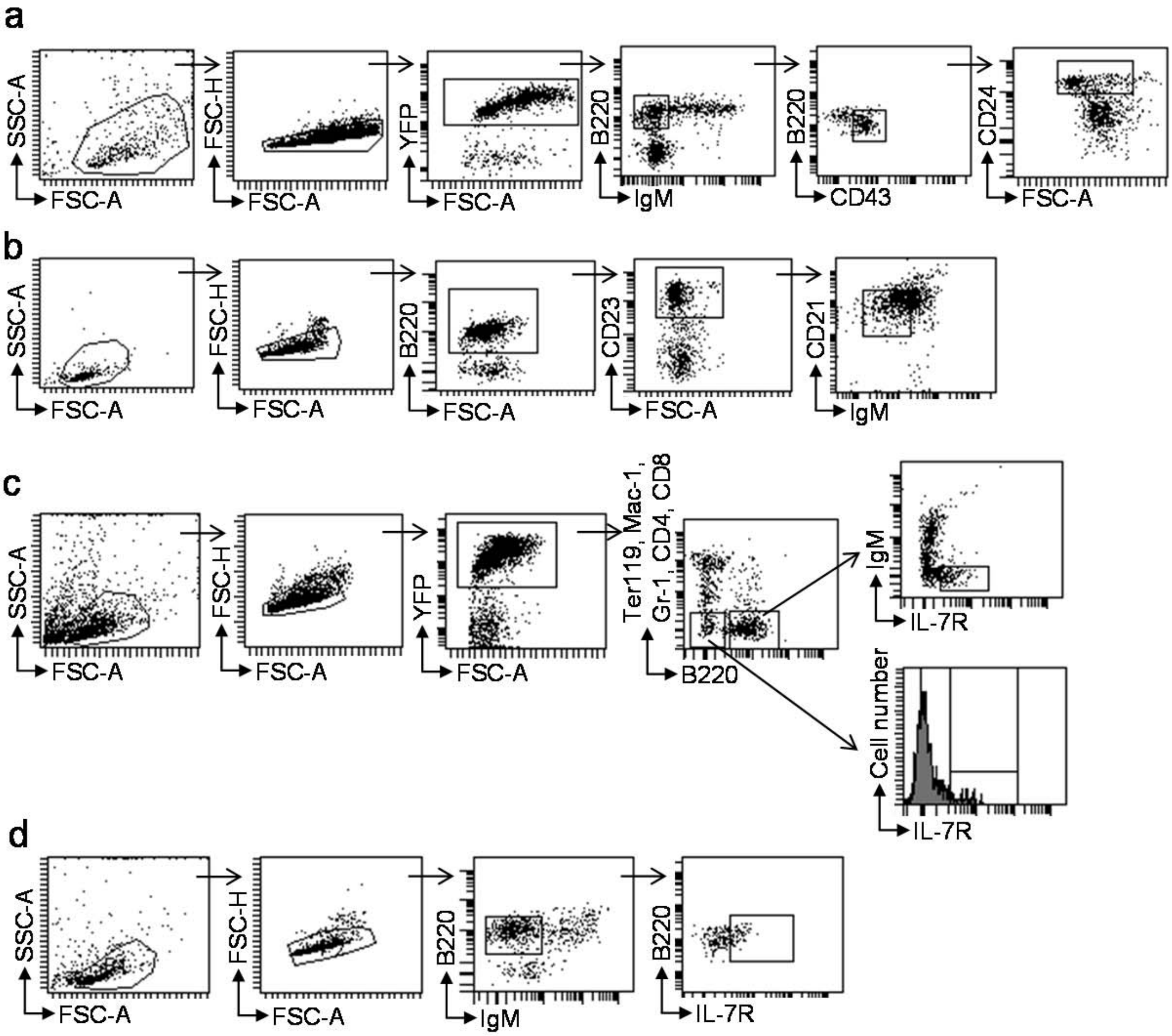

a
SSC-A
FSC-A
FSC-H
FSC-A
YFP
FSC-A
B220
IgM
B220
CD43
CD24
FSC-A
b
SSC-A
FSC-A
FSC-H
FSC-A
B220
FSC-A
CD23
FSC-A
CD21
IgM
c
SSC-A
FSC-A
FSC-H
FSC-A
YFP
FSC-A
Ter119, Mac-1, Gr-1, CD4, CD8
B220
IgM
IL-7R
Cell number
IL-7R
d
SSC-A
FSC-A
FSC-H
FSC-A
B220
IgM
B220
IL-7R

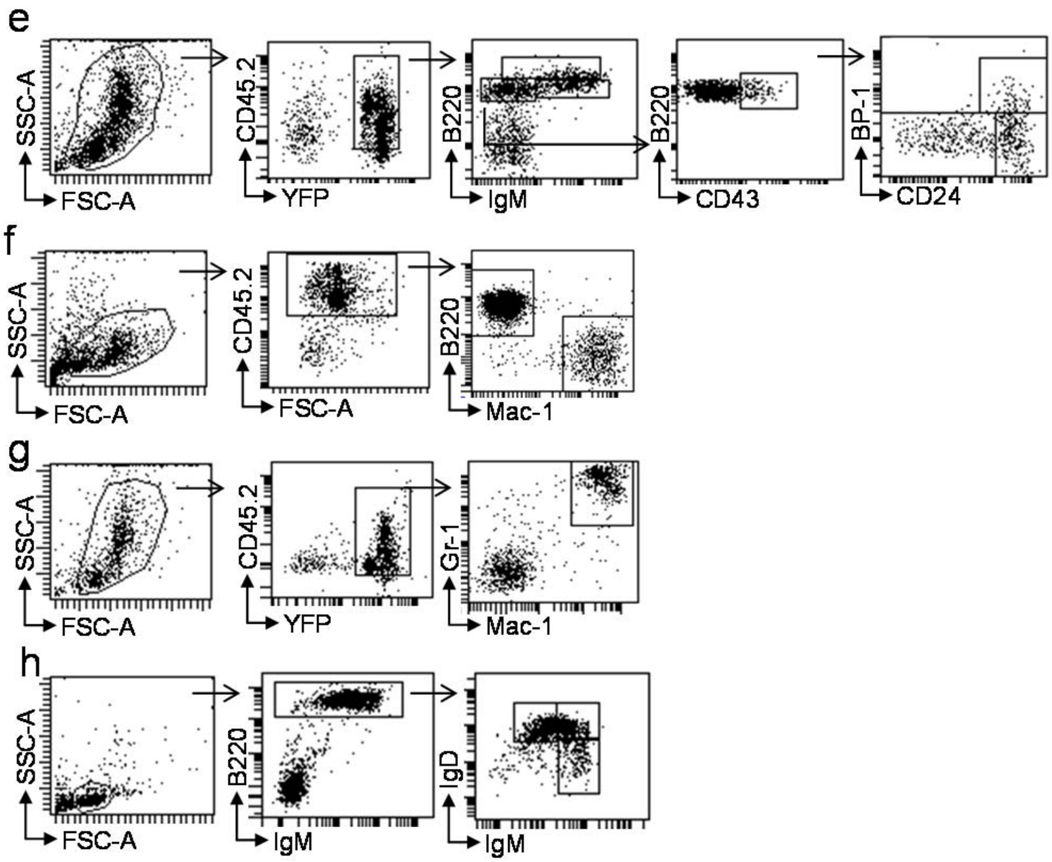


**Supplementary Figure 17.**

**Flow cytometry sorting and FACS gating strategies.** (a) Pro-B cell sort, related to Figure 1c, 2e-h, 6c, 7i, Supplementary 6a. (b) FO B cell sort, related to Figure 1c. (c) pro-B cell and CLP sort, related to Figure 4a-g, Supplementary Fig.7a,b. (d) wild-type B cell progenitor sort, related to Figure 3f-g, Figure 5c-d, Supplementary 8, Supplementary Figure 13a-b. (e) Gating strategy for flow cytometry analysis of B cell development in the bone marrow, related to Figure 1d-h, Figure 6d, Supplementary Figure 2, Supplementary Figure 15. (f) Gating strategy for flow cytometry analysis of in vitro OP9 culture, related to Figure 2a-d, g, Figure 3a-e, Figure 7c,d,f,g, Figure 8a,e,f,h,i, Supplementary Figure 5a-c, Supplementary Figure 6c-e, Supplementary Figure 12a-c. (g) Gating strategy for flow cytometry analysis of myeloid cells, related to supplementary Figure 1b-e. (h) Gating strategy for $Ca^{2+}$ flux analysis in mature B cells, related to Supplementary Figure 10b.

Fig. 1b

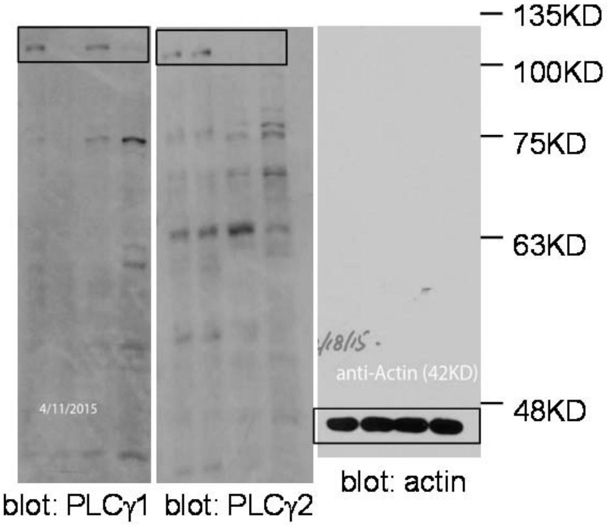
135KD
100KD
75KD
63KD
48KD
4/11/2015
anti-Actin (42KD)
blot: PLCγ1
blot: PLCγ2
blot: actin

Fig. 1c

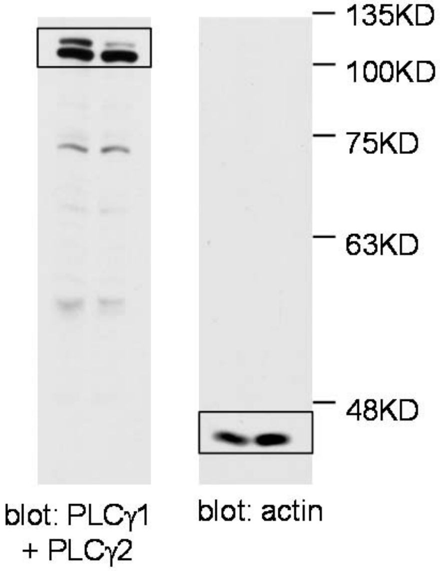
135KD
100KD
75KD
63KD
48KD
blot: PLCγ1
+ PLCγ2
blot: actin

Fig. 6a

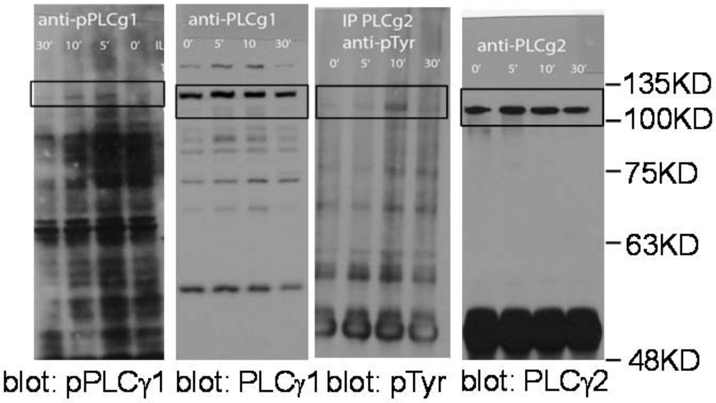


Fig. 6c

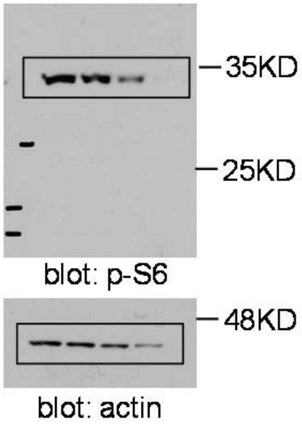


Fig. 6b

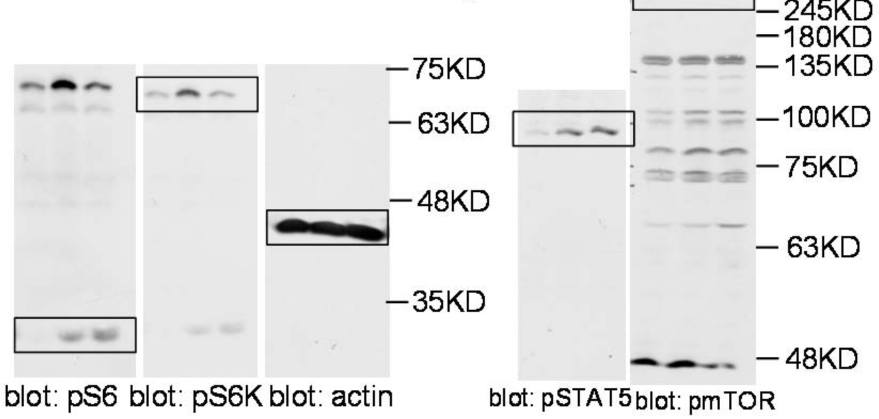


Fig. 6e

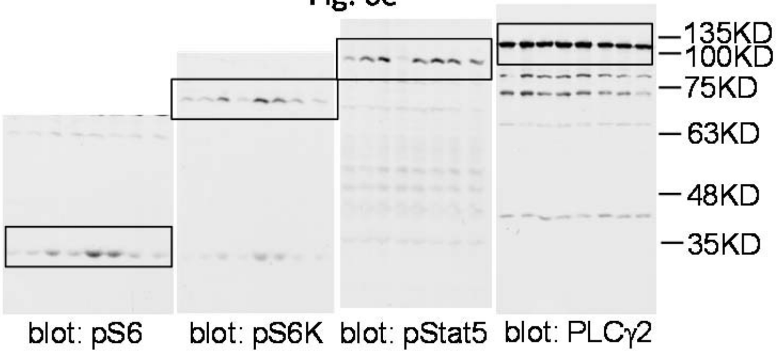


Fig. 6f

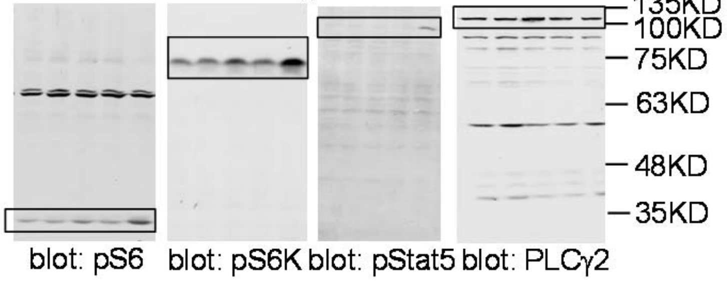

Fig. 7a

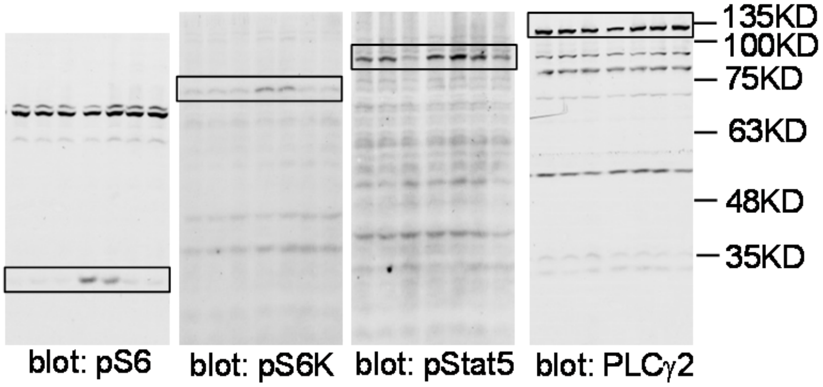


Fig. 7b

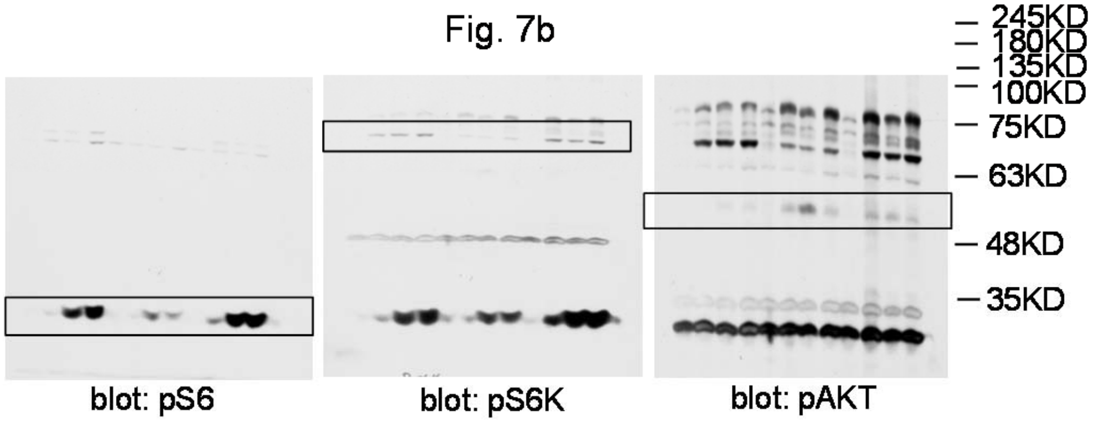


Fig. 7b

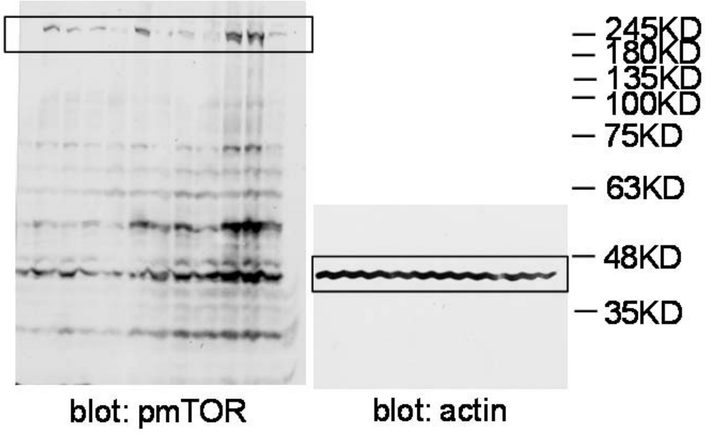

Fig. 8b

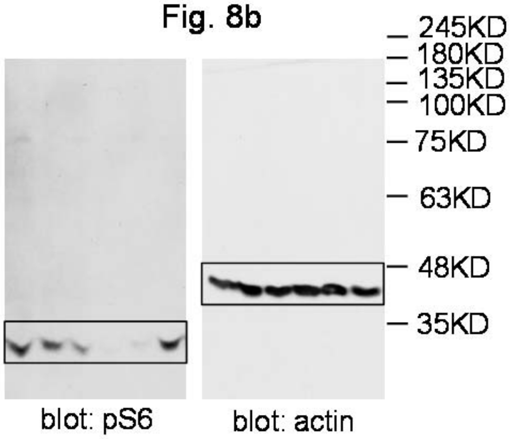


Fig. 8d

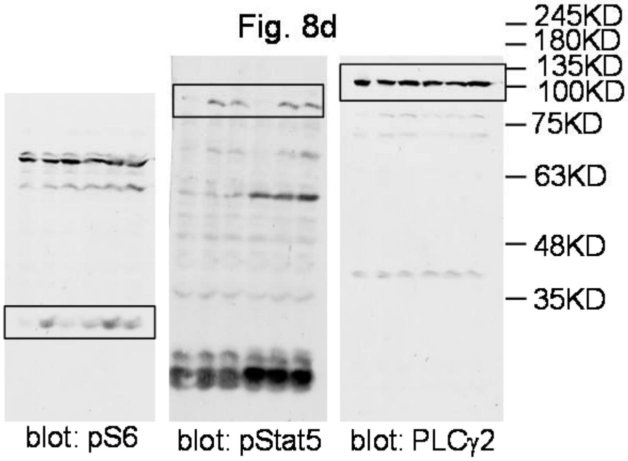


Fig. 8j

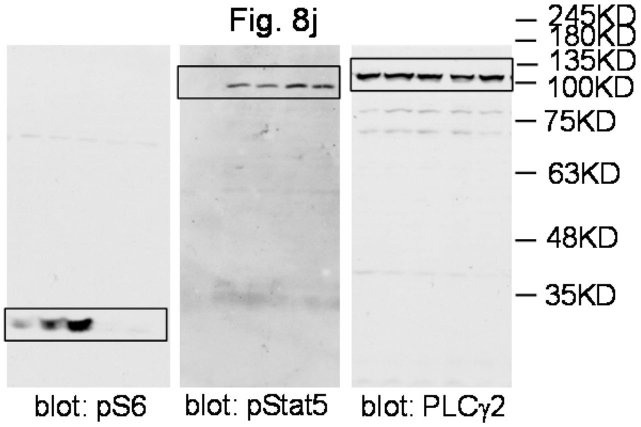

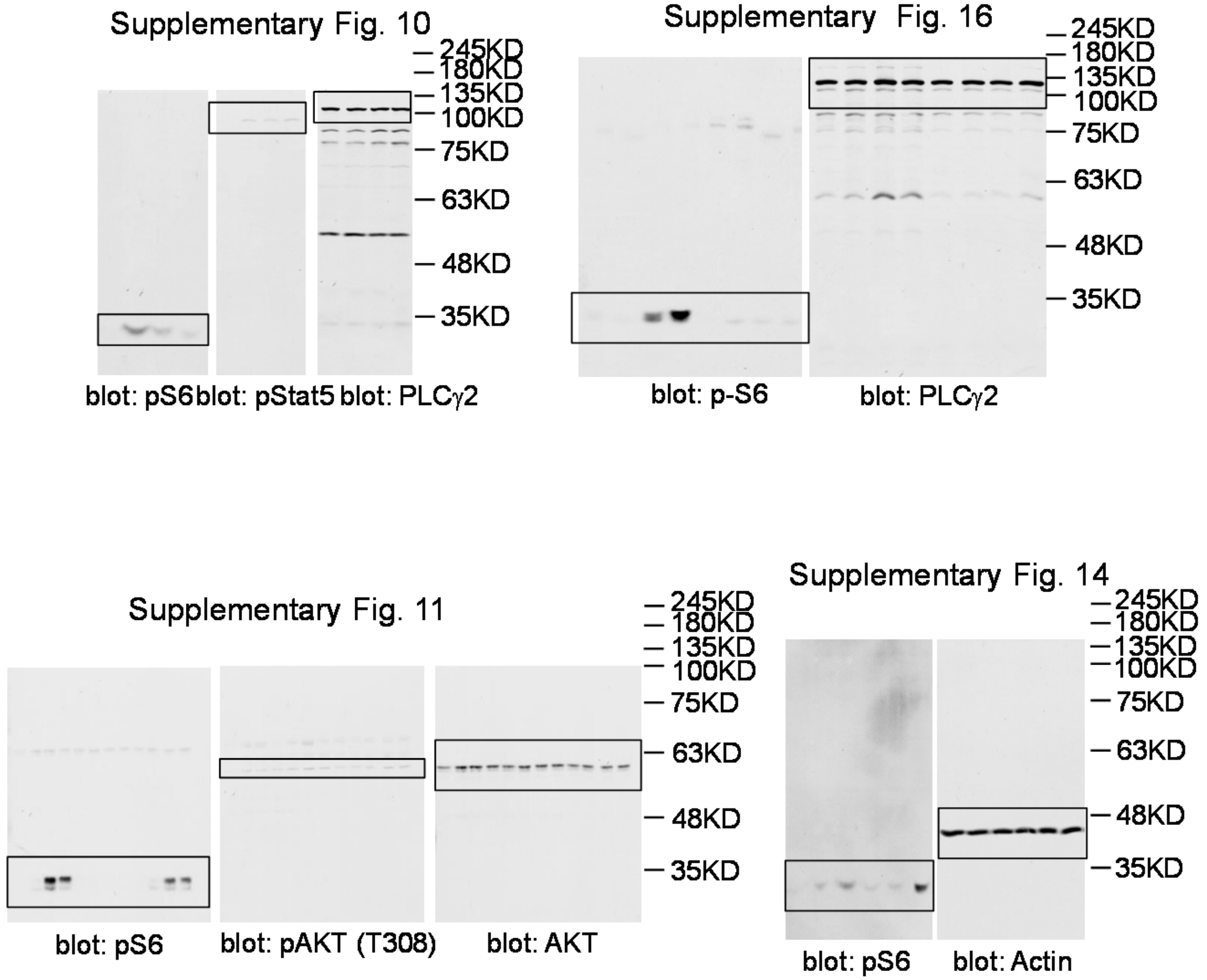


**Supplementary Figure 18. The uncropped scans of blots**
